# Fatal heart failure caused by severe pulmonary regurgitation, tricuspid regurgitation and late-onset mitral stenosis in an adult patient with Noonan syndrome: a case report

**DOI:** 10.1186/s12872-018-0878-1

**Published:** 2018-07-16

**Authors:** Yoshito Kadoya, Tsuneaki Kenzaka, Yohei Oda, Satoaki Matoba

**Affiliations:** 1 Department of Cardiovascular Medicine, Graduate School of Medical Science, Kyoto Prefectural University of Medicine, Kajii-cho 465, Kawaramachi-Hirokoji, Kamigyo-ku, Kyoto, 602-8566 Japan; 2 Division of Community Medicine and Career Development, Kobe University Graduate School of Medicine, Kobe, Japan; 3 Department of Cardiovascular Medicine, Kyotango City Yasaka Hospital, Kyoto, Japan

**Keywords:** Noonan syndrome, Heart failure, Pulmonary regurgitation, Mitral stenosis, Congenital heart disease

## Abstract

**Background:**

In patients with Noonan syndrome (NS), cardiac disorders such as pulmonary valve stenosis (PS) or hypertrophic cardiomyopathy (HCM) are common. While some patients can develop heart failure associated with HCM, the long-term outcome of adult patients with NS is reported to be good. Fatal outcomes of heart failure in patients with NS but without HCM are rare.

**Case presentation:**

We report a 25-year-old Japanese woman diagnosed with NS in adulthood. She exhibited short stature and minor facial dysmorphism and was diagnosed with PS at 1 year of age. After surgical valvuloplasty for PS at 6 years of age, her general condition became stable without specific medical treatment. She discontinued regular medical follow-up for PS. At 21 years of age, she developed acute decompensated heart failure, which was mainly right-sided heart failure due to severe pulmonary regurgitation (PR) and tricuspid regurgitation (TR). There was no evidence of HCM or PS recurrence. On the basis of the history of PS and characteristic physical features including short stature, webbed neck, and hypertelorism, she was clinically diagnosed with NS. At 25 years of age, she developed heart failure of both sides due to PR, TR and late-onset severe mitral stenosis (MS). The etiology of MS was uncertain. Owing to the patient’s condition, surgical options were considered to be extremely high risk. She was treated with optimal medical treatment as well as the occasional abdominal cavity drainage for recurrent ascites; however, she died of decompensated heart failure at 27 years of age.

**Conclusions:**

We describe an adult patient with NS without HCM who died of heart failure caused by severe PR, TR and MS. Clinicians should recognize that ongoing or late-onset cardiac disorders can develop in patients with NS, and lead to fatal heart failure. Optimal medical follow-up to monitor cardiac function and early identification of heart failure are important.

**Electronic supplementary material:**

The online version of this article (10.1186/s12872-018-0878-1) contains supplementary material, which is available to authorized users.

## Background

Noonan syndrome (NS) is a common genetic disorder with multiple congenital abnormalities, such as short stature, congenital heart disease, renal anomalies, lymphatic malformations, and characteristic facial features [1–3]. Cardiac abnormalities most notably include pulmonary stenosis (PS) and hypertrophic cardiomyopathy (HCM) [4–6]. The long-term prognosis of patients with NS depends on the severity of cardiac complications. While some patients can develop heart failure associated with HCM, the long-term outcome of adult patients with NS without HCM is reported to be good [5, 7, 8]. Here, we report a rare case of fatal heart failure due to severe pulmonary regurgitation (PR), tricuspid regurgitation (TR), and late-onset mitral stenosis (MS) in an adult patient with NS.

## Case presentation

**Fig. 1:**
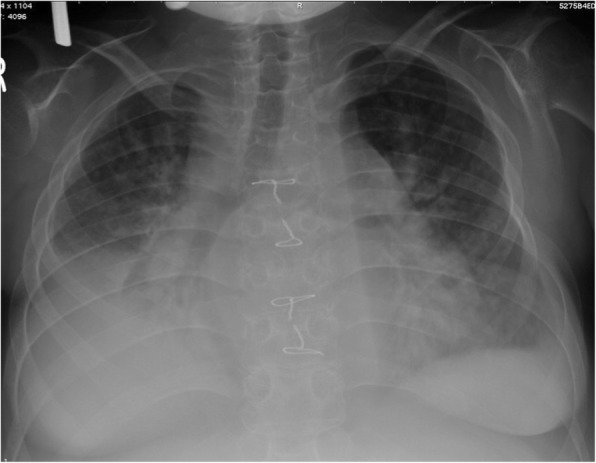
Chest radiogram showing heart enlargement with cardiothoracic ratio of 63%, pulmonary edema, and bilateral pleural effusion

**Fig. 2:**
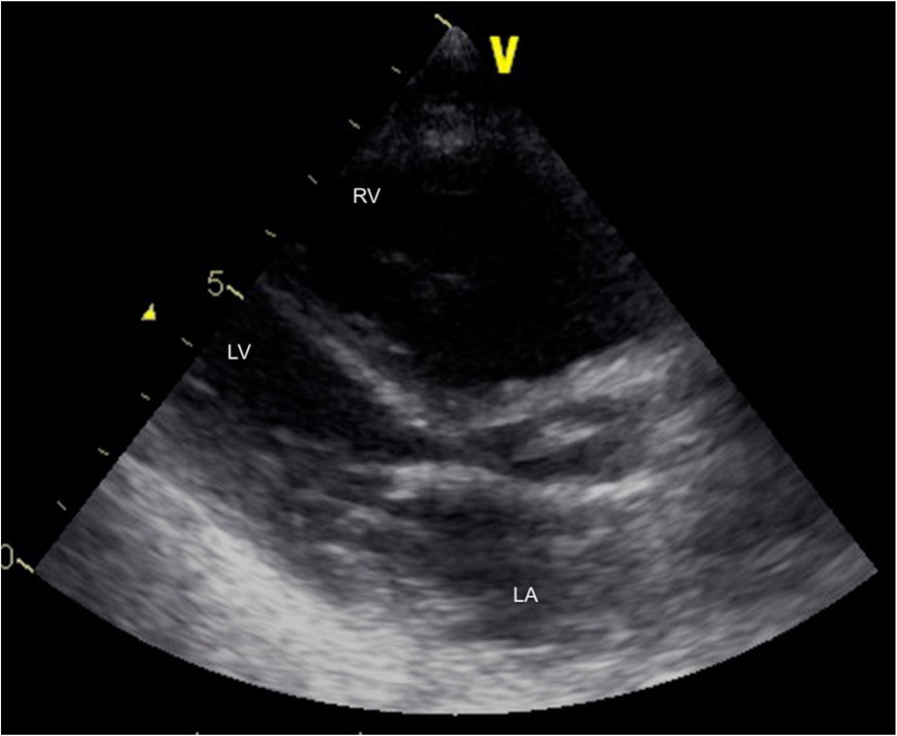
Echocardiogram in the parasternal long axis view showing the enlargement of the right-side heart and the thickening of the mitral valve leaflets. RV, right ventricle; LV, left ventricle; LA, left atrium

**Fig. 3:**
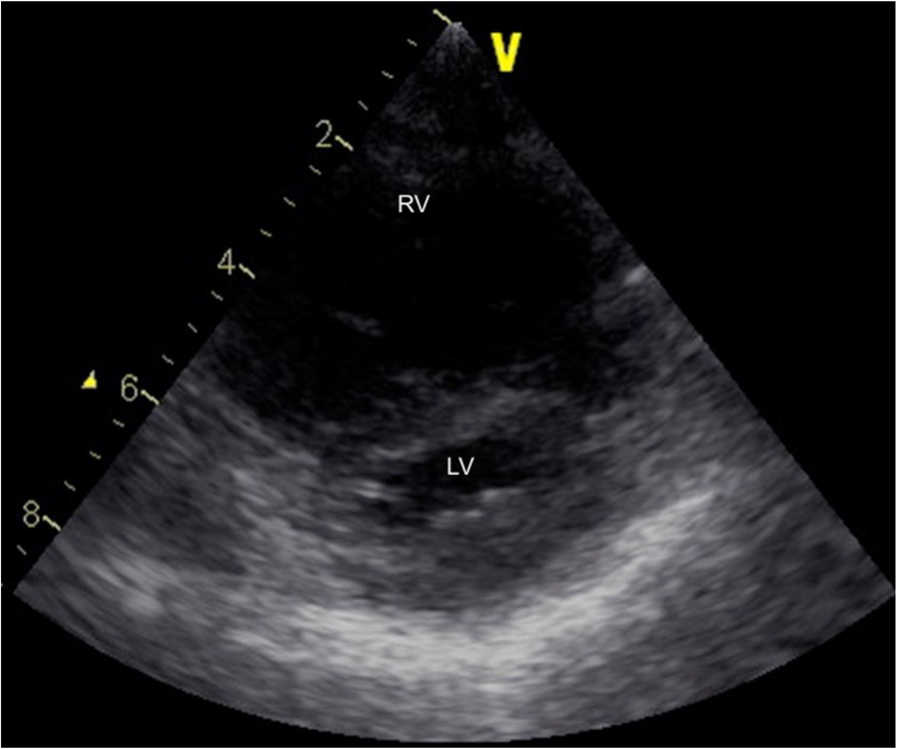
Echocardiogram in the short-axis view showing displacement of the ventricular septal wall due to the enlarged right-side heart. RV, right ventricle; LV, left ventricle

**Fig. 4:**
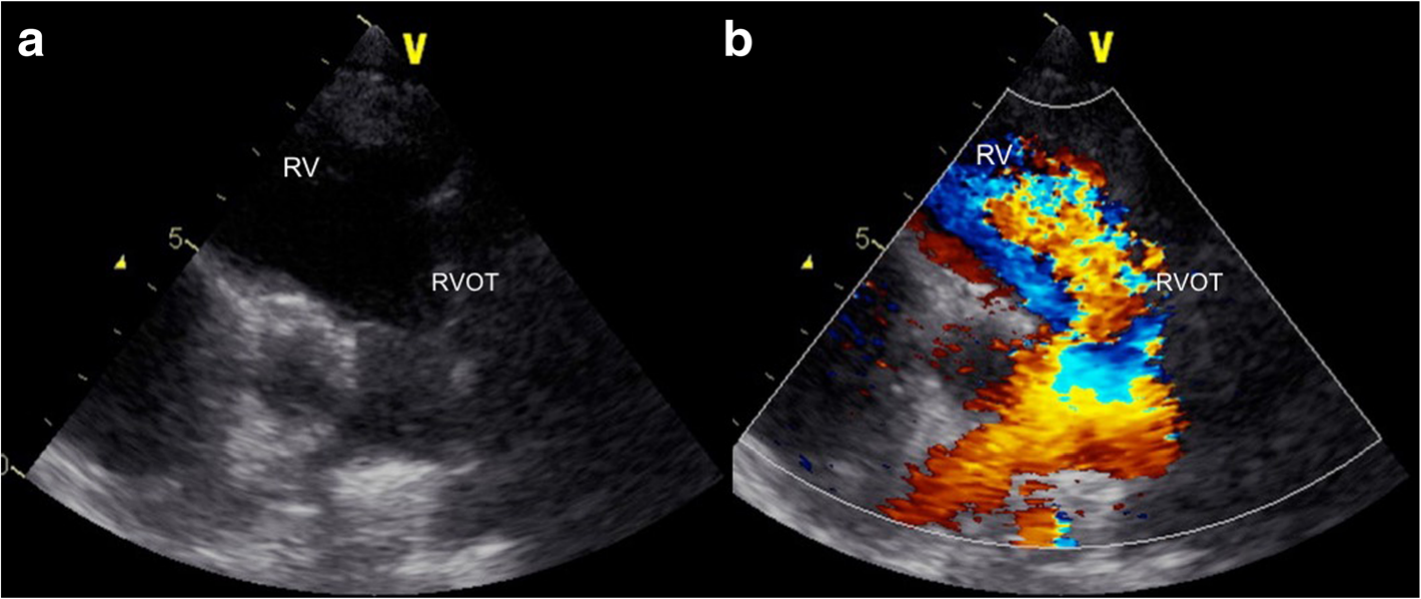
Echocardiogram in the short-axis view of aortic valve level showing severe pulmonary regurgitation. **a**: B-mode image; **b**: Color Doppler image. RV, right ventricle; RVOT, right ventricle outflow tract

**Fig. 5:**
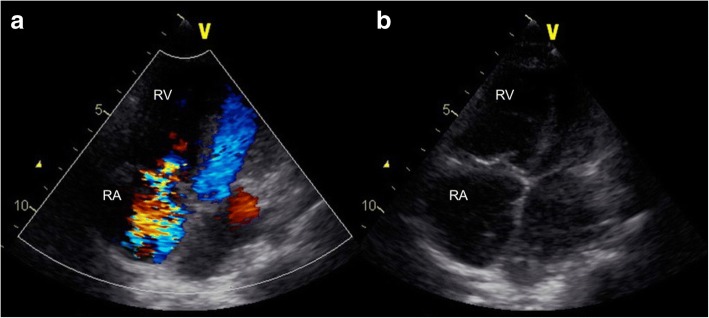
Echocardiogram in the apical view showing severe tricuspid regurgitation **a**: B-mode image, **b**: Color Doppler image. RV, right ventricle; RA, right atrium

**Fig. 6:**
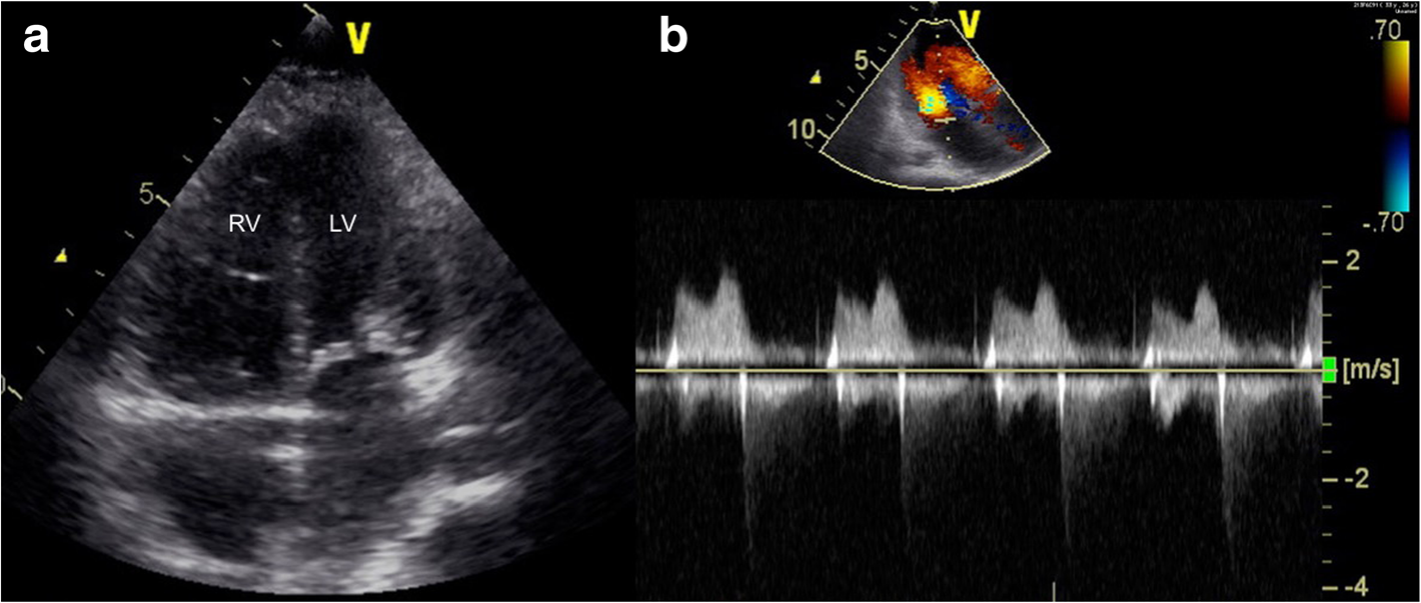
Echocardiogram in the apical view showing severe mitral stenosis with a mean pressure gradient of 10 mmHg with mild thickening of the mitral valve leaflets. **a**: B-mode image, **b**: Continuous-wave Doppler method. RV, right ventricle; LV, left ventricle


The patient was born at 39 weeks of gestation and delivered by Cesarean section due to polyhydramnios. She was 3295 g at birth. Although short stature and abnormal facial features such as depressed nose, deeply grooved philtrum, and macroglossia were recognized at birth, the signs were not associated with any particular diagnosis. At 1 year of age, she was diagnosed with pulmonary valve stenosis. At 5 years of age, a balloon valvuloplasty for severe PS was performed; however, it was not sufficient to reduce the pressure gradient of PS (from 80 mmHg to 50 mmHg). At 6 years of age, surgical valvuloplasty to enlarge the annulus and reconstruct the right ventricular outflow tract was performed, which resulted in the disappearance of the PS pressure gradient. She was followed-up at our hospital yearly. Although echocardiogram showed mild PR, her clinical condition was good without specific medical treatment. When she was a high school student, she discontinued regular medical follow-up, and started studying abroad at 18 years of age. She occasionally experienced transient leg edema during this time. At 21 years of age, she developed dyspnea, edema, and abdominal bloating. She returned to Japan; thereafter, she required an emergency hospitalization. She was diagnosed with acute decompensated heart failure, which was mainly right-sided heart failure due to severe PR and TR. It was thought that PR had been subclinically exacerbated after the surgical valvuloplasty, resulting in right-ventricular volume overload. She was also diagnosed with protein-losing enteropathy associated with abnormalities in lymphatic drainage. Echocardiography showed no evidence of HCM, MS, or PS recurrence. Cardiac catheterization revealed a normal cardiac index of 3.9 L/min/m^2^, and a normal estimated mitral valve area of 4.13 cm^2^/m^2^. On the basis of the history of PS and characteristic physical features including short stature, webbed neck, and hypertelorism, she was clinically diagnosed with NS for the first time. A chromosomal study showed 46XX with no abnormality of chromosome 12. The patient refused genetic testing. She was successfully treated with a loop diuretic, beta-blocker, angiotensin-converting enzyme inhibitor, and aldosterone inhibitor. After discharge, she resumed regular follow-up at the local hospital. Although the symptoms of heart failure, such as dyspnea and edema, persisted with a New York Heart Association class of II these symptoms could be controlled with oral medical treatment. There were no records regarding the follow-up echocardiographic findings at the local hospital. At 25 years of age, she was admitted to the local hospital again for massive ascites and marked edema and was referred to our hospital. Her height was 107 cm and her weight was 33 kg. She had a body temperature of 36.8 °C, blood pressure of 90/50 mmHg, regular pulse rate of 125 beats/min, respiratory rate of 18 breaths/min, and oxygen saturation of 95% without oxygen administration. On physical examination, she exhibited jugular venous distention at her neck, and systolic and diastolic regurgitant murmur at the left sternal border. Her breath sounds were decreased, and she had abdominal distention with no tenderness and significant leg edema. Laboratory data upon hospitalization are shown in Table 1. Chest radiography showed heart enlargement with cardiothoracic ratio of 63%, pulmonary edema, and bilateral pleural effusion (Fig. 1). Electrocardiogram showed sinus tachycardia with right axis deviation. Echocardiogram showed enlargement of the right-side heart with displacement of the ventricular septal wall, as well as severe PR, TR, and severe MS with a mean pressure gradient of 10 mmHg and mild thickening of the mitral valve leaflets (Figs. 2, 3, 4, 5 and 6, Additional file 1: Video 1, Additional file 2: Video 2, Additional file 3: Video 3, Additional file 4: Video 4). Since diastolic function based on the early diastolic mitral septal annular velocity was normal, it was unlikely that the patient had restrictive cardiomyopathy or constrictive pericarditis. Cardiac catheterization revealed a mean pulmonary artery pressure of 49 mmHg, a mean pulmonary artery wedge pressure of 33 mmHg, a left ventricular end-diastolic pressure of 24 mmHg, a low cardiac index of 1.9 L/min/m^2^, and confirmed severe MS with an estimated mitral valve area of 0.81 cm^2^/m^2^. Coronary arteries were intact. There was no evidence of rheumatic change or infectious endocarditis; the etiology of the late-onset MS was uncertain. Computed tomography of the chest and abdomen showed massive ascites as well as plural effusion and atelectasis in the right lung. On the basis of these findings, she was diagnosed with heart failure of both sides, which was mainly caused by severe PR and TR after surgical valvuloplasty for PS as well as the late-onset severe MS with uncertain etiology. Moreover, her abdominal ultrasonogram findings, including irregular external contour, enlarged left liver lobe, and splenomegaly, indicated that she had developed cirrhosis. This was thought to be associated with long-standing right-side heart failure, because of the negative viral or other hepatitis screening. Results of the analysis of ascites and plural effusion were consistent with a pure transudate. Thus, excessive fluid of the chest and abdomen was considered to have been caused by the chronic right-side heart failure in addition to the severe hypoalbuminemia associated with advanced cirrhosis and protein-losing enteropathy. In addition to the abdominal and chest drainage, fluid management using furosemide, tolvaptan, and carperitide was successfully performed. We discussed a treatment strategy with surgeons, including mitral valve replacement, tricuspid valvuloplasty, and right ventricular outflow tract reconstruction; however, considering the patient’s low left ventricular function, cirrhosis, very low albumin level, and atelectasis caused by the long-standing pleural effusion, surgical options were considered to be extremely high risk. In addition, transcatheter cardiac intervention could not be performed in Japan at that time. Therefore, we continued the optimal medication treatment as well as the occasional abdominal cavity drainage for recurrent ascites. Unfortunately, after repeated hospitalizations for ascites and edema, she died of decompensated heart failure 2 years later.

**Table 1:** Laboratory data

Parameter	Recorded value	Standard value
White blood cell count	10.5 × 10^3 /L	3.40–7.30 × 10^3 /L
Red blood cell count	3.98 × 10^6 /L	3.62–4.99 × 10^6 /L
Hemoglobin	11.7 g/dL	11.7–15.1 g/dL
Hematocrit	36.5%	34.1–45.3%
Platelet	407 × 10^3 /L	160–327 × 10^3 /L
C-reactive protein	0.60 mg/dL	≦0.20 mg/dL
Total protein	3.5 g/dL	6.4–8.4 g/dL
Albumin	1.8 g/dL	3.9–5.2 g/dL
Total bilirubin	0.23 mg/dL	0.2–1.0 mg/dL
Aspartate aminotransferase	19 U/L	12–35 U/L
Alanine aminotransferase	13 U/L	6–33 U/L
Lactate dehydrogenase	226 U/L	114–243 U/L
Alkaline phosphatase	274 U/L	120–362 U/L
γ-glutamyltranspeptidase	57 U/L	3–54 IU/L
Creatinine	0.20 mg/dL	0.30–1.10 mg/dL
Sodium	137 mEq/L	138–150 mEq/L
Potassium	4.4 mEq/L	3.6–5.0 mEq/L
Glucose	99 mg/dl	98–109 mg/dl
B-type natriuretic peptide	39.1 pg/mL	< 18.4 pg/mL
Total cholesterol	156 mg/dL	125–220 mg/dL
Glucose	102 mg/dL	65–110 mg/dL
Thyroid stimulating hormone	3.647 μIU/mL	0.350–4.940μIU/mL
Free-T3	1.97 pg/mL	1.71–3.71 pg/mL
Free-T4	0.87 ng/dL	0.70–1.48 ng/dL
Type IV collagen 7S	9.2 ng/mL	< 6.0 ng/mL
Hyaluronic acid	97 ng/mL	< 50 ng/mL
Prothrombin time	12.4 s	10.2–14.0 s

**Additional file 1: Video 1.** Echocardiogram in the parasternal long axis view showing enlargement of the right-side heart and the thickening of the mitral valve leaflets. (MP4 768 kb)



**Additional file 2: Video 2.** Echocardiogram in the short-axis view showing displacement of the ventricular septal wall due to the enlarged right-side heart. (MP4 735 kb)



**Additional file 3: Video 3.** Color Doppler echocardiogram in the short-axis view at the aortic valve level showing severe pulmonary regurgitation. (MP4 790 kb)



**Additional file 4: Video 4.** Color Doppler echocardiogram in the apical view showing severe tricuspid regurgitation. (MP4 770 kb)


## Discussion and conclusions

We encountered a fatal case of heart failure, which was caused by severe PR and TR after surgical valvuloplasty for PS, and late-onset severe MS, in an adult patient with NS. Our experience revealed two important clinical issues. Ongoing or late-onset cardiac disorders can develop in patients with NS and lead to fatal heart failure. Optimal medical follow-up to monitor cardiac function and early identification of heart failure are important to facilitate the appropriate management and treatment of patients with NS.

Ongoing or late-onset cardiac disorders can develop in patients with NS, and lead to fatal heart failure. The most common cardiac abnormalities associated with NS are PS (50–60%) and HCM (20%) [4–6]. The existence of HCM has been reported to be the important risk factor for death in patients with NS [9–11]. Although data regarding the long-term outcomes in patients with NS are scarce, several reports suggest that the long-term survival rate is good in patients without HCM [5, 7, 8]. Colquitt et al. reported that the mortality rate in patients with NS was 7.1% during a mean follow-up period of 14 years [7]. In that study, only 1.0% of patients died of heart failure, and all deaths were associated with HCM. Thus, fatal outcomes from heart failure in patients with NS but without HCM are presumed to be rare. In patients with NS, however, long-term cardiac follow-up is recommended because of ongoing cardiac disorders requiring the treatment of heart failure or arrhythmias [12]. Cardiac complications in adult patients with NS include mild aortic insufficiency, substantial right ventricular outflow tract obstruction, or restrictive cardiomyopathy [13, 14]. In our patient, PR had been subclinically exacerbated after surgical valvuloplasty, and subsequently right ventricular dysfunction developed in adulthood. In addition, late-onset severe MS had developed, although its etiology was unclear. The potential risk of cardiac disorders should be recognized in adult patients with NS, even if the patient is clinically stable.

Optimal medical follow-up to monitor cardiac function and the early identification of heart failure are important to facilitate the appropriate management and treatment of patients with NS. Approximately one-third of patients with NS and PS require therapeutic interventions, such as percutaneous balloon valvuloplasty or open surgical valvuloplasty [5]. Although little is known about the prognosis during the post-operative period of PS in patients with NS, long-term survival is believed to be good based on a previous long-term follow-up study [5, 9]. In fact, our patient had maintained good function after a valvectomy for PS. Unfortunately, she did not receive regular postoperative follow-up for more than 5 years. During this period, heart failure subclinically progressed and, at hospitalization, the patient’s condition had deteriorated irreversibly. Furthermore, because of the patient’s critical condition, including low left ventricular function, cirrhosis, very low albumin level, and atelectasis caused by the long-standing pleural effusion, surgical options were not possible. If her cardiac status had been closely evaluated on a regular basis, the early identification and subsequent treatment of heart failure may have been possible with surgical options.

As the recent innovation in radical surgery for congenital heart disease has shown high success and high survival rates in neonatal patients, the number of cases of adults with congenital heart disease are increasing. However, the long-term results of many types of radical surgery for congenital heart disease remain unclear. Thus, long-term follow-up of patients with congenital heart disease, including patients with NS, has become increasingly important. Optimal medical follow-up should be conducted for all post-operative patients with congenital heart disease, even for those in good clinical condition.

In conclusion, we describe an adult patient with NS without HCM who had heart failure in both sides that was caused by severe PR and TR after surgical valvuloplasty for PS, and late-onset severe MS. Clinicians should recognize that ongoing or late-onset cardiac disorders can develop in these patients, leading to fatal heart failure, even in the absence of HCM. Optimal medical follow-up to monitor cardiac function and the early identification of heart failure are thus crucial.

## Abbreviations

HCM: Hypertrophic cardiomyopathy
MS: Mitral stenosis
NS: Noonan syndrome
PR: Pulmonary regurgitation
PS: Pulmonary stenosis
TR: Tricuspid regurgitation
